# Relationship between bone turnover and left ventricular function in primary hyperparathyroidism: The EPATH trial

**DOI:** 10.1371/journal.pone.0173799

**Published:** 2017-04-13

**Authors:** Nicolas Verheyen, Astrid Fahrleitner-Pammer, Evgeny Belyavskiy, Martin R. Gruebler, Hans Peter Dimai, Karin Amrein, Klemens Ablasser, Johann Martensen, Cristiana Catena, Elisabeth Pieske-Kraigher, Caterina Colantonio, Jakob Voelkl, Florian Lang, Ioana Alesutan, Andreas Meinitzer, Winfried März, Helmut Brussee, Burkert Pieske, Stefan Pilz, Andreas Tomaschitz

**Affiliations:** 1 Department of Cardiology, Medical University of Graz, Graz, Austria; 2 Department of Internal Medicine, Division of Endocrinology and Metabolism, Medical University of Graz, Graz, Austria; 3 Medizinische Klinik mit Schwerpunkt Kardiologie, Campus Virchow-Klinikum, Charité-Universitaetsmedizin Berlin, Berlin, Germany; 4 Swiss Cardiovascular Center Bern, Department of Cardiology, Bern University Hospital, Bern, Switzerland; 5 Hypertension Unit, Internal Medicine, Department of Experimental and Clinical Medical Sciences, University of Udine, Udine, Italy; 6 Department of Physiology, University of Tübingen, Tübingen, Germany; 7 Clinical Institute of Medical and Chemical Laboratory Diagnostics, Medical University of Graz, Graz, Austria; 8 Synlab Academy, Synlab Services LLC, Mannheim, Germany; 9 Medical Clinic V (Nephrology, Hypertensiology, Endocrinology), Medical Faculty Mannheim, Ruperto Carola University Heidelberg, Mannheim, Germany; 10 Department of Health Sciences and the EMGO^+^ Institute, VU University Amsterdam, Amsterdam, The Netherlands; 11 Specialist Clinic for Rehabilitation Bad Gleichenberg, Bad Gleichenberg, Austria; University of Adelaide, AUSTRALIA

## Abstract

Observational studies suggested a link between bone disease and left ventricular (LV) dysfunction that may be pronounced in hyperparathyroid conditions. We therefore aimed to test the hypothesis that circulating markers of bone turnover correlate with LV function in a cohort of patients with primary hyperparathyroidism (pHPT). Cross-sectional data of 155 subjects with pHPT were analyzed who participated in the “Eplerenone in Primary Hyperparathyroidism” (EPATH) Trial. Multivariate linear regression analyses with LV ejection fraction (LVEF, systolic function) or peak early transmitral filling velocity (e’, diastolic function) as dependent variables and N-terminal propeptide of procollagen type 1 (P1NP), osteocalcin (OC), bone-specific alkaline phosphatase (BALP), or beta-crosslaps (CTX) as the respective independent variable were performed. Analyses were additionally adjusted for plasma parathyroid hormone, plasma calcium, age, sex, HbA1c, body mass index, mean 24-hours systolic blood pressure, smoking status, estimated glomerular filtration rate, antihypertensive treatment, osteoporosis treatment, 25-hydroxy vitamin D and N-terminal pro-brain B-type natriuretic peptide. Independent relationships were observed between P1NP and LVEF (adjusted β-coefficient = 0.201, P = 0.035) and e’ (β = 0.188, P = 0.042), respectively. OC (β = 0.192, P = 0.039) and BALP (β = 0.198, P = 0.030) were each independently related with e’. CTX showed no correlations with LVEF or e’. In conclusion, high bone formation markers were independently and paradoxically related with better LV diastolic and, partly, better systolic function, in the setting of pHPT. Potentially cardio-protective properties of stimulated bone formation in the context of hyperparathyroidism should be explored in future studies.

## Introduction

Left ventricular (LV) systolic and diastolic dysfunction account for high morbidity and mortality and often occur in concert with disturbances in bone metabolism. Accumulating evidence suggests a direct link between bone disease and LV function that may be pronounced in primary hyperparathyroidism (pHPT). In a bone biopsy study conducted in patients with chronic heart failure (CHF) the local bone environment showed evidence of dysregulated and catabolic state that was correlated with circulating biomarkers of bone turnover [1]. In 60 CHF patients bone mineral density was inversely related with parathyroid hormone (PTH) and an independent predictor of a composite outcome of death, implantation of a LV assist device, or inotrope dependency [2]. Vertebral fractures are present in up to 16% of chronic heart failure (CHF) patients [3, 4] and the risk of hip fracture is almost doubled in CHF patients when compared to patients without CHF [5]. Vice versa, Pfister and colleagues recently reported that low bone mineral density (BMD) was an independent predictor of new-incident CHF in a large cohort of apparently healthy subjects followed for a mean of 9.9 years, even after adjustment for cardiovascular risk factors [6].

PHPT is a condition of autonomously and chronically elevated PTH [7]. Patients with pHPT show biochemical features of stimulated bone turnover [8] and abnormal bone microstructure [9, 10] and this leads to an increased risk of osteoporosis and fractures [11]. Emerging evidence supports the role of high PTH as a novel risk factor for heart failure [12, 13]. In fact, pHPT patients often present with alterations in LV function [14, 15], besides their altered bone metabolism, and are exposed to an increased risk of cardiovascular mortality [16]. PHPT may therefore constitute a suitable human model to study the potential crosslink between bone turnover and LV function. Yet, studies in humans are completely lacking.

We therefore aimed to test the hypothesis that bone turnover markers are associated with LV systolic and diastolic function in a cohort of pHPT patients.

## Methods

### Patients and setting

This research was approved by the Ethics Committee of the Medical University of Graz (# 24–031 ex 11/12). All subjects provided written informed consent.

Cross-sectional data from screening participants for the single-center, randomized, placebo-controlled, double-blind, parallel-arm “Eplerenone in Primary Hyperparathyroidism” (EPATH) Trial were used in the present analyses. The EPATH trial tested the effect of daily eplerenone application over 8 weeks in comparison to placebo on PTH levels in subjects with pHPT [17]. Further details on the study design and setting have been previously reported [17, 18]. Main inclusion criteria for trial participation were age of at least 18 years and informed consent, main exclusion criteria were any acute illness or any disease with an estimated life expectancy less than one year or ongoing radiation or chemotherapy. For the present analyses, we included all participants with a biochemical diagnosis of pHPT (n = 155). All subjects provided written informed consent. The study was approved by the local ethics committee and complies with the Good Clinical Practice (GCP) guidelines and the Declaration of Helsinki.

### Laboratory, echocardiography and ambulatory blood pressure monitoring

Blood samplings were performed during the morning (07:00AM—11:00AM) after an overnight fast. Patients remained in the seated position before blood sampling for at least 10 minutes. All samples were kept at room temperature before analyses, except for those that were used to measure PTH which were kept at 4°C. A pre-specified volume of blood samples was centrifuged and filled into 1mL aliquots and frozen at -80°C. Markers of osteoblast activity (N-terminal propeptide of procollagen type 1 (P1NP), total osteocalcin [OC], bone-specific alkaline phosphatase [BALP]) and osteoclast activity (beta-crosslaps [CTX]) were determined from one time frozen serum on an IDS-iSYS multi-discipline automated system using Enzyme-Linked Immunosorbent Assays (Immunodiagnostic Systems Ltd., Boldon, UK). Intra- and interassay coefficients of variation (CV) are 3.2% to 9.6% and 5.5% to 9.5% for P1NP, 1.3% to 2.2% and 2.7% to 5.1% for OC, 2.6% to 6.5% and 3.7% to 6.4% for BALP and 1.7% to 3.0% and 2.5% to 10.9% for CTX. PTH was measured immediately after blood sampling on an Elecsys 2010 (Elecsys immunoassay analyzer, Cobas, Roche Diagnostics GmbH, Mannheim, Germany) applying an ElectroChemiLuminescence ImmunoAssay (ECLIA). Normal range is 15–65 pg/ml (1.6–6.9 pmol/L), intra- and interassay CVs are 1.5% to 2.7% and 3.0% to 6.5%. Plasma calcium was adjusted for hypoalbuminemia as previously reported [19]. Other laboratory parameters were assessed using routine laboratory methods as previously described [17].

Echocardiographic examinations were performed with a Vivid 7 or Vivid 9 (GE Healthcare, Chalfont St Giles, UK), as previously reported [19]. All images and recorded loops were analyzed in a central core lab (Echocardiography CoreLab, Department of Cardiology, Medical University of Graz, Graz, Austria) by a single investigator who was blinded to individual participant data (EB). Tissue Doppler imaging was employed to measure peak early filling velocities of the mitral septal and lateral ring (e’, in cm/s). Following international guidelines averaged e’ was generated according to the formula e’_average_ = (e’_medial_ + e’_lateral_)/2 [20]. LV ejection fraction (LVEF) was calculated by Simpson biplane method of disks [21].

A validated portable device for continuous ambulatory blood pressure monitoring (ABPM) (Mobil-O-Graph, I.E.M. GmbH, Stolberg, Germany) was employed to execute the 24-hours ABPM.

### Primary hyperparathyroidism

Hypercalemic pHPT was defined as hypercalcemia (total serum albumin adjusted calcium > 2.55 mmol/L) and inappropriately high PTH of > 46 pg/mL at the study baseline visit [17, 22, 23]. Normocalcemic pHPT was defined as PTH > 65 pg/mL, albumin adjusted plasma calcium above the median of the reference range (>2.35 mmol/L) and plasma ionized calcium within normal ranges, in the absence of advanced chronic kidney disease (eGFR < = 40 mL/min/m2) or 25-hydroxy vitamin D (25(OH)D) deficiency (25(OH)D < 20 ng/dL) as potential causes for secondary hyperparathyroidism [17, 22–24].

### Statistical methods

Assuming a correlation co-efficient of 0.225 lead to a sample size of 153 subjects to achieve a power of 80% at a significance level of 5%.

Continuous variables are expressed as the mean +/- standard deviation (SD) or as the median (interquartile range, IQR) as appropriate. Categorical variables are expressed as numbers (percentages). The distribution of continuous parameters and their residuals were evaluated by test of Kolmogorov-Smirnov, kurtosis, skewness, concordance between the mean and median, and visual inspection. Non-normally distributed variables were 10-logarithmized, squared (only LVEF) or Ln-logarithmized (only CTX), as appropriate, before use in parametrical procedures. Associations between bone turnover markers with PTH, calcium, LVEF and e’ were assessed using Pearson correlation analyses. In stepwise multivariate linear regression models with each of the bone markers as a respective independent variable, model 1 included as additional independent covariates PTH and calcium. In model 2, further adjustment was made for parameters that are considered classical modifiers of either bone turnover or cardiac function. These included age, sex, HbA1c, body mass index (BMI), mean 24-hours systolic blood pressure (SBP), smoking status, estimated glomerular filtration rate (eGFR, CKDEPI), ongoing antihypertensive treatment, ongoing osteoporosis treatment, serum 25(OH)D, and N-terminal pro-brain B-type natriuretic peptide (NT-proBNP). Stepwise multivariate linear regression models with LVEF or e’ as the respective dependent variable and P1NP, OC, BALP, or CTX as a respective independent variable were performed. Further adjustments followed model 1 and model 2 as described above. For all stepwise multivariate linear regression analyses, a P-value < 0.05 was used for inclusion and a P-value > 0.1 was used for exclusion of variables.

Normocalcemic pHPT may potentially differ from hypercalcemic pHPT with regard to an interaction between bone turnover and LV function. Therefore, all multivariate linear regression analyses were repeated exclusively in subjects with normocalcemic and with hypercalcemic pHPT, respectively.

For statistical analyses we used SPSS 22.0 (SPSS, Inc, Chicago, IL). A two-sided P-value < 0.05 was considered statistically significant.

## Results

Mean age was 67.0 +/- 10.6 years and 122 subjects (78.7%) were females. Median PTH was 99.1 pg/mL (81.5–124.6) and mean adjusted calcium was 2.63 +/- 0.14 mmol/L. PHPT was hypercalcemic in 113 subjects (72.9%). Baseline characteristics are shown in Table 1.

**Table 1:** Baseline characteristics of 155 subjects with primary hyperparathyroidism.

**General**	Age, years	67.0 +/- 10.6
	Females, n (%)	122 (78.7%)
	Body mass index, kg/m^2^	27.8 +/- 5.0
	Smokers, n (%)	59 (38.1%)
	Estimated GFR, mL/min/1.73m^2^	76.9 +/- 16.4
	Glycated hemoglobin A1_C_, mmol/mol	37.0 (35.0–40.0)
	Total Cholesterol, mg/dL	203.0 +/- 42.3
**Calcium metabolism**	Hypercalcemic pHPT, n (%)	113 (72.9%)
	Parathyroid hormone (1–84), pg/mL	99.1 (81.5–124.6)
	25-hydroxy vitamin D, ng/mL	34.3 +/- 11.4
	Adjusted calcium, mmol/L	2.63 +/- 0.14
	Phosphate, mg/dL	2.42 +/- 0.37
**Bone**	P1NP, ng/mL	48.0 (31.2–71.3)
	Bone-specific alkaline phosphatase, μg/mL	19.1 (14.7–25.8)
	Osteocalcin, ng/mL	22.8 (16.0–35.1)
	Beta-crosslaps, ng/mL	0.44 (0.23–0.71)
	Osteoporosis treatment, n (%)	24 (15.5%)
	RANKL-inhibitor	7 (4.5%)
	Bisphosphonates	16 (10.3%)
	Other	1 (0.6%)
**Cardiovascular**	Left ventricular ejection fraction, %	63.0 (59.0–67.7)
	e’ (average), cm/s	7.1 +/- 2.3
	Mean 24-hours SBP, mmHg	125.5 +/- 12.2
	Mean 24-hours DBP, mmHg	76.4 +/- 8.6
	Antihypertensive medication (yes/no), n (%)	83 (53.5%)

Parameters are expressed as mean +/- SD or median (interquartile range), as appropriate.

Abbreviations: GFR, glomerular filtration rate (CKD EPI); pHPT, primary hyperparathyroidism; RANKL, receptor activator of nuclear factor kappa-B ligand; P1NP, procollagen type 1 amino-terminal propeptide; SBP, systolic blood pressure; DBP, diastolic blood pressure.

### Bone markers and PTH/calcium

In multivariate regression analyses (model 2), PTH was directly and independently correlated with P1NP (adjusted β-coefficient = 0.210, P = 0.013), OC (β = 0.321, P = 0.001), BALP (β = 0.270, P = 0.002) and CTX (β = 0.378, P = 0.001). Calcium was directly and independently correlated with P1NP (β = 0.180, P = 0.036) and BALP (β = 0.182, P = 0.038), but not with OC (β = 0.105, P = 0.210) and CTX (β = 0.065, P = 0.440). Details are shown in Table 2.

**Table 2:** Correlations between parathyroid hormone/calcium and markers of bone turnover in univariate and multivariate analyses.

Dependent variable	Explanatory variable	Pearson correlation analysis		Multivariate linear regression analysis			
				Model 1		Model 2	
		r	P	β	P	β	P
P1NP	PTH	0.145	0.073	0.149	0.074	0.210	0.013
	Calcium	0.299	0.001	0.268	0.002	0.179	0.035
OC	PTH	0.271	0.001	0.298	0.001	0.321	0.001
	Calcium	0.199	0.014	0.108	0.193	0.105	0.210
BALP	PTH	0.259	0.001	0.236	0.004	0.270	0.002
	Calcium	0.308	0.001	0.243	0.003	0.182	0.038
CTX	PTH	0.339	0.001	0.318	0.001	0.378	0.001
	Calcium	0.249	0.002	0.131	0.128	0.065	0.440

Model 1 includes calcium and parathyroid hormone as covariates. Model 2 additionally includes age, sex, HbA1c, body mass index, mean 24-hours systolic blood pressure, smoking status, estimated glomerular filtration rate (CKDEPI), ongoing antihypertensive treatment, ongoing osteoporosis treatment, 25-hydroxy vitamin D, and N-terminal pro-brain B-type natriuretic peptide.

β represents adjusted regression coefficient.

Abbreviations: P1NP, N-terminal propeptide of procollagen type 1; OC, N-mid osteocalcin; BALP, bone-specific alkaline phosphatase; CTX, beta-crosslaps; PTH, parathyroid hormone.

### Bone markers and LV function

With adjustment for PTH and calcium (model 1), P1NP was independently related with e’ (β = 0.212, P = 0.021) and LVEF (β = 0.182, P = 0.043). OC was significantly related with e’ (β = 0.264, P = 0.004), but not with LVEF (β = 0.149, P = 0.100). BALP was significantly related with e’ (β = 0.191, P = 0.042), but not with LVEF (β = 0.165, P = 0.072). CTX showed a significant correlation with e’ (β = 0.217, P = 0.022), but not with LVEF (β = 0.121, P = 0.197).

After multivariate adjustment (model 2), P1NP remained an independent determinant of e’ (β = 0.188, P = 0.042) and LVEF (β = 0.201, P = 0.035). Both OC and BALP were independently related with e’ (OC: β = 0.192, P = 0.039; BALP: β = 0.198, P = 0.030). CTX was neither correlated with e’ (β = 0.146, P = 0.133) nor with LVEF (β = 0.102, P = 0.310). Detailed results are shown in Table 3.

**Table 3:** Correlations between markers of bone turnover and echocardiographic parameters of left ventricular function in univariate and multivariate analyses.

Dependent variable	Explanatory variable	Pearson correlation analysis		Multivariate linear regression analysis			
				Model 1		Model 2	
		r	P	β	P	β	P
e‘	P1NP	0.190	0.021	0.212	0.021	0.188	0.042
	OC	0.224	0.006	0.264	0.004	0.192	0.039
	BALP	0.147	0.076	0.191	0.042	0.198	0.030
	CTX	0.168	0.047	0.217	0.022	0.146	0.133
LVEF	P1NP	0.077	0.347	0.182	0.043	0.201	0.035
	OC	0.061	0.460	0.149	0.100	0.138	0.158
	BALP	0.072	0.381	0.165	0.072	0.108	0.248
	CTX	0.085	0.314	0.121	0.197	0.102	0.310

Model 1 includes calcium and parathyroid hormone as covariates. Model 2 additionally includes age, sex, HbA1c, body mass index, mean 24-hour systolic blood pressure, smoking status, estimated glomerular filtration rate (CKDEPI), ongoing antihypertensive treatment, ongoing osteoporosis treatment, 25-hydroxy vitamin D, and N-terminal pro-brain B-type natriuretic peptide.

β represents adjusted regression coefficient.

Abbreviations: P1NP, N-terminal propeptide of procollagen type 1; OC, N-mid osteocalcin; BALP, bone-specific alkaline phosphatase; CTX, beta-crosslaps; LVEF, left ventricular ejection fraction.

### Hypercalcemic versus normocalcemic primary hyperparathyroidism

Multivariate regression analyses (model 2) were repeated exclusively in subjects with hypercalcemic pHPT (n = 113). P1NP did not significantly correlate with e’ (β = 0.196, P = 0.098) or LVEF (β = 0.129, P = 0.261). OC and BALP were independently related with e’ (OC: β = 0.260, P = 0.031; BALP: β = 0.221, P = 0.044), but not with LVEF (OC: β = 0.116, P = 0.328; BALP: β = -0.025, P = 0.814). There was no correlation between CTX and e’ or LVEF, respectively.

None of the bone markers correlated with e’ or LVEF when analyses were restricted exclusively to subjects with normocalcemic pHPT (n = 42).

## Discussion

In a well-characterized and relatively large cohort of subjects with pHPT, circulating markers of bone formation were independently related with e’ as a measure of LV diastolic function. P1NP was also independently correlated with LVEF. These associations were independent of potentially confounding parameters. Thus, our data provide novel evidence on a direct link between bone formation and LV function in the setting of pHPT.

Clinical data on a link between bone turnover and LV function are sparse and longitudinal studies reporting associations with cardiovascular outcome data have been inconsistent. In a small cohort of 20 hypertensive patients without pHPT, higher P1NP was not associated with echocardiographic parameters of diastolic dysfunction [25]. High CTX was consistently associated with increased cardiovascular mortality in clinical studies [26–28]. By contrast, longitudinal studies on bone formation markers reported arbitrary results, with most showing an inverse association between bone markers and mortality [26, 28, 29]. Only one study is available that aimed at examining potential effects of PTH application on LV function. These authors analyzed the progress of levels of NT-proBNP in osteoporotic subjects one, three and six months after initiation of treatment with PTH (1–34) and found no change in comparison to baseline [30].

Bone formation markers were independently related with PTH levels in our cohort and correlations between these markers and LV function were pronounced after adjustment for PTH and calcium. Moreover, these associations were only present in subjects with hypercalcemic disease. Our observations should therefore be regarded in the context of autonomously elevated PTH. Besides skeletal impairment, alterations in LV function and an increased cardiovascular risk have been documented in pHPT, even in mild disease [14–16]. Epidemiological studies suggested a mechanistic crosslink between bone and cardiac disease [1–6] and it is reasonable to assume that this relationship could be pronounced in pHPT. Surprisingly, our data rather point towards cardio-protective properties of stimulated bone formation. In that aspect the emerging role of bone metabolism for endothelial functioning, systemic energy metabolism and stem cell proliferation and mobilization may be of relevance. Recent studies have provided evidence on a molecular crosstalk between bone forming and endothelial cells facilitating a close interweavement of endothelial function and bone turnover [31]. By promoting the secretion of vascular endothelial growth factor (VEGF) and nitric oxide from endothelial cells PTH is a stimulator of the bone-vascular axis in experimental settings [32, 33]. Some studies indicated that these PTH actions on bone and on the vasculature impose beneficial effects to the cardiovascular system. In rats with induced myocardial infarction PTH application led to mobilization of angiogenic progenitor cells from bone marrow and improved survival and reduced infarct size compared to sham treatment, probably via promoting VEGF-mediated cardiac neovascularization [34]. Also in humans, PTH administration was associated with increases in circulating hematopoietic stem cells and an improvement in carotid intima-media thickness [35, 36]. Patients with pHPT exhibited higher numbers of circulating bone marrow derived progenitor cells compared to healthy controls, and increased VEGF levels [37]. It could therefore be speculated that in pHPT high bone formation markers can be considered a surrogate of preserved responsiveness of the bone-vascular axis to PTH.

Our observations could be explained by direct effects of osteoblast-derived hormones on LV function. Particularly the role of OC for cardiovascular disease has recently received attraction. OC is secreted from osteoblasts, e.g. upon activation by PTH, and is increasingly considered to play a pivotal role in systemic energy metabolism. Low OC levels were associated with chronic heart failure [38] and an increased risk of cardiovascular events [39]. Nevertheless, potential direct effects of OC on cardiomyocytes remain unclear and should be addressed in future research. Vice versa, LV function may have direct influence on bone formation. In fact, implantation of a ventricular assist device in patients with CHF was associated with an increase in P1NP suggesting that improvement in LV function may stimulate bone formation [40].

Several aspects of the present work can be considered as significant strengths. These include the novelty aspect, as—to the best of our knowledge—no clinical study has yet examined a link between bone turnover markers and LV function in pHPT. Correlations with LV diastolic function were consistent using different formation markers and the relatively large sample size enabled us to adjust comprehensively for important potentially confounding parameters. Moreover, laboratory and echocardiographic parameters were measured prospectively and under blinded and standardized conditions.

Our study is limited by its cross-sectional character, and no conclusions can be drawn on a potential cause-effect relationship between bone formation and LV function. In addition, due to the single-center design our results may not be applicable to other pHPT populations. Despite the use of multivariate regression models residual confounding cannot be ruled out.

As a conclusion, circulating markers of bone formation were independently related with LV diastolic function and, partly, with systolic function in a relatively large-sized cohort of patients with pHPT. These data provide first clinical evidence on a direct link between bone formation and LV function in pHPT. Potentially cardio-protective properties of stimulated bone formation in the context of hyperparathyroidism and underlying mechanisms should be explored in future studies.

## Supporting information

S1 File SPSS file that contains all parameters used for analyses in this work. (SAV)
